# Evaluation of one-month foundation course for the first year undergraduate students at a Medical College in Puducherry, India

**DOI:** 10.30476/jamp.2020.86857.1272

**Published:** 2020-10

**Authors:** DEEPIKA VELUSAMI, AMOL R DONGRE, RAJENDRA N KAGNE

**Affiliations:** 1 Department of Physiology, Sri Manakula Vinayagar Medical College and Hospital, Kallitherthalkuppam, Madagadipet, Puducherry- 605 107, India; 2 Department of Community Medicine, Sri Manakula Vinayagar Medical College and Hospital, Kallitherthalkuppam, Madagadipet, Puducherry-605 107, India; 3 Department of Forensic Medicine, Sri Manakula Vinayagar Medical College and Hospital, Kallitherthalkuppam, Madagadipet, Puducherry-605 107, India

**Keywords:** Medical students, Medical teaching, Medical college

## Abstract

**Introduction:**

Medical Council of India has revised the undergraduate medical curriculum by introducing “Competency-based Medical Education” which emphasizes the foundation course of one-month duration. This period is said to be essential for students to get acclimatized to the new college environment. The present study evaluated the first one-month foundation course from students and faculty members’ point of view.

**Methods:**

The present study was program evaluation. The study participants were all 150 first year medical students joining the college and preclinical department faculty in the academic year, 2019-20. The foundation program was pre-planned and implemented as per the Medical Council of India guidelines. The program was evaluated using a pre-designed questionnaire where the items were aligned with the research question and inputs were obtained from all faculty members. Kirkpatrick framework level 1 was used for evaluation. Feedback was received from the faculty members by force field analysis and from student’s using a five-point Likert scale. A summative approach to the qualitative content analysis was done to identify certain themes from the text data and infer meaning for the force field analysis obtained from the faculty. Considering the high rating for most of the sessions, we arbitrarily considered values above 75% to reflect good consensus and below 75% to reflect poor consensus. Consensus measure expressed in percentage was obtained for each item. The quantitative data were analyzed using open Epi info version 7.0.

**Results:**

The consensus scores ranged from 73.7 to 83.3 percent. The sessions on learning styles, student support system, self-directed learning, communication skills, medical ethics, soft skills, and orientation to health systems in India reflected good consensus, indicating that these sessions were well received by the students. Other sessions like stress management, interpersonal skills, presentation skills, email writing and ethics for mobile usage reflected poor consensus, implying the need for further improvement. As per the faculty perception, good coordination, teamwork, and proper planning at interdepartmental and intradepartmental levels were the key features for the successful implementation of the course.

**Conclusion:**

Overall, the sessions in the foundation course were well received by the students. As felt by both students and faculty, more interactive sessions need to be incorporated. The major strength of the course was the skill module, visit to special school, and field visit to the community and primary health center. The findings will help us to improve our next year foundation program to meet the purpose of the Foundation course.

## Introduction

Medical Education in India is being revolutionized with the introduction of “Competency- based undergraduate curriculum” for the Indian Medical Graduate (IMG) ( 1 ). It marks a significant shift from the teacher centered learning to student centered learning, which stresses the practicalities, skills development, medical ethics, and better doctor-patient relationship. The newly revised curriculum insists on the foundation course of one month duration at the beginning of the MBBS program with an objective to sensitize the fresh medical students with the required knowledge and skills that will assist them in acclimatizing and making them familiar to the new professional environment. As envisaged by the Medical Council of India (MCI), an IMG should have attributes like Clinician, Lifelong Learner, Leader, Communicator, and Professional ( 2 ). The foundation course helps the students to get an overview of the MBBS curriculum and learn medicine effectively to achieve the required attributes.

The purpose of the Foundation Course has been to: a) Orient the students to all aspects of the medical college environment; b) Equip them with basic important, skills required for patient care and enhance their communication, language, computer and learning skills; c) Provide an opportunity for peer and faculty interactions and an overall sensitization to various learning methodologies ( 3 ). One of the initial requirements of any successful program is learner satisfaction and continuous quality improvement; hence, the regulating body expects the colleges to undertake evaluation and development in the program. Therefore, the present study aimed to evaluate the first foundation course at Sri Manakula Vinayagar Medical College and Hospital, Pondicherry using the students and faculty members’ point of view. 

## Methods

The present study was conducted in Sri Manakula Vinayagar Medical College and Hospital, located in Kallitherthalkuppam, Madagadipet, Puducherry, India. It is a private Medical College affiliated to Pondicherry University. It admits 150 students every year and holds a good record of academic performance profile for the last twelve years. 

It was a program evaluation at Kirkpatrick level 1 ( 4 ). At level 1, the purpose was to determine the satisfaction of the learners to the course offered. 

The study participants were all students joining first year Bachelor of Surgery and Bachelor of Medicine (MBBS) undergraduate course (n=150; male=70, female=80) for the academic year 2019-20; also, all pre-clinical faculty members (n=13; Male=4, female=9) lecturing in basic sciences departments, that is Anatomy (n=5), Physiology (n=5) and Biochemistry (n=3), were included in the study. 

The overall program was coordinated by the Dean of college. To begin with, Medical Education Unit (MEU) organized the “Curriculum Implementation and Support Program (CISP)” for the faculty members of pre-clinical department for planning, implementing and evaluating the new curriculum. The focus of the CISP was on four curricular strategies: foundation course, early clinical exposure, integrated teaching, and skills training. Later, all pre-clinical department faculty played a key role in outlining the schedule and planning for the foundation course. This was in alignment with the MCI guidelines ( 5 ). The schedule was approved by the Curriculum Committee and displayed it on the college website ( 6 ). Afterwards, a couple of meetings were organized to finalize the resource persons, allocating the sessions, framing the session objectives, and discussion on the sessions planned by the resource persons. The faculty were encouraged to use various teaching learning methods such as lectures, interactive sessions, role plays, small group discussions, interviews, reflective writing, presentations, and debate to meet the session objectives and thereby program goals. The faculty were instructed to plan their session and discuss with the dean the feasibility and resources required for their respective session. For selected topics like soft skills, children with special needs and Yoga, external resource persons were invited to lead the sessions. In collaboration with the Engineering College faculty, the planning of the content to be delivered regarding language and computer skills was carried out. 

The newly joined students underwent one-month foundation course on the MCI guidelines ( 3 ). Student medical health check-up was done, and they were immunized for Hepatitis B. The various modules covered included Orientation module (36hrs); Skill module (38hrs); Field visit to Community Health Centers (8hrs); Professional development and Ethics (41hrs); Sports, Extracurricular activities and Yoga (23hrs); and Enhancement of Language and Computer skills (40hrs). The program was conducted in the large auditorium with flexible seating arrangements for large group teaching and small group activities. 

*Orientation Module:* The students were made aware of the goals, roles and competencies of the Indian Medical graduate, the new MCI curriculum plan, anti-ragging committee, rules and regulations of the college, evaluation of their learning skills and student support system. The students were sensitized to the learning strategies, principles of family practice, medical ethics, history of Medicine and alternate systems in India.

*Skill module:* The students were divided into four batches (n=18 or 19) and rotated for learning various skills. The skills training sessions conducted were: first aid, basic life support, communication skills workshop, chemical and fire safety, documentation in medical records, universal precautions, and waste management workshop. The sessions were facilitated by skilled clinicians who had prior experience of running such skills-based training in conferences of medical students.

*Field visit to Community and Primary Health Center:* The students were divided into four batches (n=18 or 19) and taken to Primary Health Centre as part of their orientation to our health care delivery system. In addition to exposure visit, they had sessions on national health goals, health policies, and public health problems in India.

*Professional development and ethics:* External resource team was arranged that briefed interactively on interpersonal skills, presentation skills, email writing, ethics for mobile usage, leadership skills, time management, and coping with stress. The sensitization session on disability competence was organized in coordination with Sathya Special School, Puducherry. The students were taken to Sathya Special School in batches and were made to interact with teachers and staff of school. They were sensitized to the skills and attributes essential to provide health care to patients with disabilities. The students were sensitized to group dynamics, pedagogy, self-directed learning, ethics in medical practice, the value of integrity, honesty and respect in medical field, concepts of sympathy and empathy, consequences of ethical and unethical professional behavior with the help of role plays by intern batch, roles and responsibilities of the doctor, rights and responsibilities of the patient, the realities and art of medical practice, carrier pathways in the medical field, and the white coat ceremony. 

*Sports, Extracurricular Activities and Yoga:* Sports and extracurricular activities were organized by the sports and Cultural Committee, along with the student council. These activities laid down a sound platform to exhibit their talents and made them understand the importance of physical fitness and teamwork. Yoga sessions were organized for 9hrs in support with external resource person.

*Language and Computer skills:* These training sessions were organized in coordination with the other institution. The computer skills involved hands-on training for Microsoft office word, excel, PowerPoint presentation, internet operation, and recent trends and applications in Medical use. The language skills involved listening, speaking, reading, writing, body language, and Tamil basics for students from other states.

**Feedback from Students:** On the last day of foundation course, all students were requested to fill out the feedback form. It was a pre-designed and pre-defined, 5-point Likert scale type questionnaire ( 7 ). Since it was a program evaluation, we aligned the items in the questionnaire with the research questions to ensure the internal consistency. It was reviewed by all the authors to make sure that the instrument measures what it is supposed to measure ( 8 ). The items in the questionnaire were based on foundation course module and competencies stated by MCI. In order to avoid social desirability and ensure anonymity, we did not collect information such as name and roll numbers. 

The quantitative data were analyzed using open Epi info version 7.0 (Centers for Disease Control and Prevention, Altanta, USA) ( 9 ). A consensus measure, expressed in percentage, was obtained for each of the items. It is a newer method for analyzing the Likert Scale. Values at the upper end of the range indicated more "agreement" than values at the lower range. A value closer to 1.0 or 100% has less dispersion around the weighted mean value and indicates greater agreement ( 10 ). Low consensus values were identified through high dispersion around the mean value. Considering the high rating for most of the sessions, we arbitrarily considered values above 75% to reflect good consensus and below 75% as to reflect poor consensus. The first author collected and entered the data. The second author reviewed it and discrepancies were sorted out through discussion. 

**Feedback from the faculty:** Feedback was received from faculty by Force Field Analysis (FFA) after the course completion ( 11 ). The review meeting was conducted on the same day of completion of the foundation course facilitated by a trained person with all pre-clinical department faculty (n=13) who had been the part of the foundation course. FFA was employed to uncover the areas that worked well and areas for improvement. Initially, they were asked to individually enlist “What went well in the foundation course?” and “What are the areas for improvement?” Next, all 13 faculty were given an opportunity to freely talk about their perceptions and express their views on the foundation course. At last, the results were compiled and presented. The students’ feedback was collected on the final day of the foundation course through a questionnaire using a five-point Likert scale, Kirkpatrick model 1. The overall feedback was evaluated, and the report was generated.

A summative approach to the FFA was undertaken to find certain themes from the text data and infer meaning. Statements with the same meaning were grouped together and the data were classified for and against perceptions ( 11 ).

**Ethical consideration:** Since the feedback was obtained as a part of the program, a waiver of consent was obtained from the institutional ethics committee (Ethical Clearance number: SMVMCH-ECO/AL/389/2019).

## Results

All the 150 students responded for the feedback. Of the 150 students, 70 (46.67%) and 80 (53.33%) were male and female, respectively. The mean age of the students was 18.14±0.79 years. Students responded positively about all aspects of the foundation course. The consensus values ranged from 73.7% to 83.32%. 

Table 1 represents the mean and consensus scores of the students’ perceptions on learning styles, stress management, and student support system. The sessions on learning styles (75.42%), student support system (77.45%), and self-directed learning (75.72%) had good consensus scores. The session on stress management (74.59%) got poor consensus.

**Table 1:** Learning styles, stress management and student support system

Domain	SA n (%)	A n(%)	NADA n(%)	D n(%)	SD n(%)	Mean	Consensus (%)
The session on learning skills and styles made me aware of the learning strategies.	69(46)	67(44.67)	13(8.67)	0(0)	1(0.67)	1.66	75.42
The session on stress management was useful.	75(50)	63(42)	9(6)	2(1.33)	1(0.66)	1.61	74.59
I am aware of the Student Support System.	88(58.66)	53(35.33)	8(5.33)	1(0.66)	0(0)	1.48	77.45
The session on self directed learning was useful.	69(46%)	71(47.33)	10(6.67)	1(0.66))	1(0.66)	1.65	75.72

SA: Strongly agree; A: Agree; NADA: Neither agree nor disagree; D: Disagree; SD: Strongly disagree

Table 2 represents the student’s sensitization to professionalism, ethics, and communication. The sessions on the development of leadership skills, conflict management and time management (75.88%), communication skill (75.86%) and medical ethics (77.96%) represented good consensus. The session on interpersonal skills, presentation skills, email writing, and ethics for mobile usage (73.70%) reflected poor consensus.

**Table 2:** Sensitization to professionalism, ethics, and communication

Domain	SA n(%)	A n(%)	NADA n(%)	D n(%)	SD n(%)	Mean	Consensus (%)
The session on interpersonal skills, presentation skills, email writing and ethics for mobile usage were helpful.	67(44.67)	63(42)	19(12.67)	3(2)	0(0)	1.72	73.70
The session on development of leadership skills, conflict management and time management were helpful.	77(51.33)	58(38.67)	15(10)	0(0)	0(0)	1.59	75.88
The session on communication skill made me to understand the importance of communication skill in health care system.	98(65.33)	40(26.67)	11(7.33)	0(0)	1(0.66)	1.44	75.86
I am sensitized to the concept of Medical ethics.	82(54.67)	61(40.67)	6(4)	1(0.66)	0(0)	1.51	77.96

SA: Strongly agree; A: Agree; NADA: Neither agree nor disagree; D: Disagree; SD: Strongly disagree

Table 3 represents the students’ orientation to the health systems in India. The sessions of history of medicine and alternate medicine (75.46%), principles of family practice (77.71%), National Goals and policies in India (77.44%), and special school and field visits (83.32%) reflected good consensus.

**Table 3:** Orientation to health system in India

Domain	SA n(%)	A n(%)	NADA n(%)	D n(%)	SD n(%)	Mean	Consensus (%)
I am sensitized to the history of Medicine and various alternate Medicine practices in India.	60(40)	68(45.33)	21(14)	1(0.67)	0(0)	1.75	75.46
I learnt the principles of family practice.	53(35.33)	80(53.33)	15(10)	2(1.33)	0(0)	1.77	77.71
I am sensitized to understand the National Health Goals and Policies in India.	50(33.33)	80(53.33)	17(11.33)	3(2)	0(0)	1.82	77.44
The visit to special school and field visit were useful.	112(74.67)	35(23.33)	2(1.33)	1(0.66)	0(0)	1.28	83.32

SA: Strongly agree; A: Agree; NADA: Neither agree nor disagree; D: Disagree; SD: Strongly disagree

The session on disability competence reported the greatest consensus (83.32%); followed by the session on medical ethics (77.96%) and communication skills (75.86%). Table 4 represents the areas of improvement. According to the students, sessions like documentation lecture, ethics session, games, extracurricular activities, and sports needed improvement. Students stated that activity- based sessions can be incorporated wherever possible. As to the faculty members’ point of view, sessions like anti-ragging, interpersonal skills, mobile usage, walk around campus, and skill module needed further improvement in planning the sessions and making them more interactive. Both student and faculty perceived that the Yoga, computer, and language sessions should be improved further and hands-on training session should be included. 

**Table 4:** Areas for improvement

Student’s perspectives	Faculty perspectives
Sessions that need improvement	Sessions that need improvement
-Theory on chemical and fire safety can be improved	-Anti ragging
-Documentation lecture -(4)	-Interpersonal relationship
-Yoga sessions were theory based-(23)	-Policy on mobile usage
-Ethics session could have been short-(4)	-Yoga sessions
-Interpersonal skills and mobile usage	-Computer sessions can be related to Medicine
-Computer sessions need be well planned- (28)	-Field visit and walk around campus should not be on same day
-More games and Extra-curricular can be planned-(9)	-Tamil sessions can be increased
-Sports to be well planned-(5)	-For skill modules, the group size can be increased
-Tamil learning skills should be emphasized	
Other issues	Other issues
-Some faculty used Tamil only, so difficult for other state students	-More workshops and activities to be planned rather than theory
-Circular regarding rules and regulations can be given	-Google groups and WhatsApp group for faculty
-Crowded sessions	-Better venue place
-Some sessions/points were repeated	-Duration can be reduced
-Lecture sessions were boring-(14)	-Disciplined to be maintained strictly
-More interactive sessions needed -(8)	
-Course duration can be shortened	

## Discussion

Overall, all the sessions were well received by the students. The sessions on learning styles, student support system, self-directed learning, professional development, ethics, communication skills, and orientation to health systems in India were well received by the students, while sessions like stress management, interpersonal skills, presentation skills and email writing needed improvement. Both students and faculty felt that the one-month course duration can be reduced. They also reported that Yoga and Computer sessions required further improved session planning and they emphasized incorporating more interactive sessions in small groups. This was due to the reason that Yoga session was more theory-based and less time was allotted for practical sessions. Regarding the computer sessions, students felt that the topics discussed during those sessions provided them with only basic knowledge that they were already familiar with. 

A study carried out by Mittal et al., in 2013, evaluated the student’s perception on “foundation program” carried out for two days for the second year MBBS students, reporting that the majority of the students graded the overall program as a very good and useful program. No student responded as unsatisfactory ( 12 ). Similarly, the one day foundation program conducted for fresh MBBS entrants was evaluated and rated as good ( 13 ) based on students and faculty perspectives. Evaluations of the three day orientation program conducted in a medical college in Kerala revealed that the students had positive approach towards the program and had benefited from it ( 14 ).

The sessions on learning styles, student support system, self-directed learning, development of leadership skills, conflict management, time management, communication skills, medical ethics, and students’ orientation to the health systems were well received by the students. The reasons reflected from their session plans were that these sessions facilitated small group discussions, and had ice-breaking activities, team building activities, planned for short durations, field visits, energizing activities, hands-on training, motivating the students to present their ideas, creativity and reflections. Active learning made the students responsible for their learning process and developed higher-order thinking ( 15 - 17 ).

 On the contrary, sessions like stress management, interpersonal skills, presentation skills, Yoga, computer skills and email writing were not well received by the students and from faculty perspectives. These sessions were more of teacher-ecentered mode, lengthy PowerPoint presentation lectures were given, and students were passive learners. The students (n=14) stated that these sessions were boring and lacked interactions. For addressing a larger group, though lectures are convenient, but students lack the opportunity to process the perceived information (18-20).

The strength of the foundation course was the successful implementation and evaluation of the one-month course for all 150 students. All the students filled out the questionnaire. Good teamwork, planning and co-operation among the faculty were the crux factors for the successful implementation of this program. Since the feedback was taken on the last day of the program, whether students were able to recall all sessions fully and give feedback is doubtful.

Since MCI had made one-month foundation course mandatory in all medical colleges of India, the foundation course organized in our medical college would provide an idea for other medical colleges about the planning, implementation, and evaluation process. The course can be followed as per the MCI guidelines in other colleges with contextual modifications as per their circumstances keeping in mind that this period is employed effectively by the students to be acclimatized with the new professional atmosphere.

The present evaluation gave an idea of the advantages as well as barriers towards the implementation and the evaluation of foundation course. This will help us to improve the next foundation course for medical undergraduates. This will, in turn, help us to organize the forthcoming foundation course in an effective way. More follow-up evaluation is required to check the achievements of desired goals of the program. However, the limitation of the present evaluation should be kept in mind. As it was an evaluation study, the findings are specific to context. We measured the lower outcome of the framework and in future it would be interesting to measure the effect of this program on the students’ behavior at workplace as a professional in the future. 

## Conclusion

Overall, the foundation course was well received by the students and faculty. The program evaluation paved way to explore the strengths and weakness of various sessions. Also, the students were sensitized to the competencies and goal of IMG at the beginning of the course itself. In future, follow up studies should be conducted to measure the effect of this program on the students’ behavior at real work setting. 

Disclosure: The authors declare no conflict of interest. No funding was received for this study.
